# An evaluation of coronary atherosclerosis using coronary CT in subjects with asymptomatic carotid lesions

**Published:** 2014-04-08

**Authors:** Vito Bonomo, Davide Piraino, Umberto Marcello Bracale, Salvatore Evola, Mariaconcetta Di Piazza, Claudia Vicari, Ambra Lupo, Giuseppe Inga, Giuseppe Andolina, Pasquale Assennato, Salvatore Novo

**Affiliations:** 1 Department of Internal Medicine, Cardiovascular and Nephrologic Diseases, Department of Cardiology, Cardiovascular Diseases School of Specialization, University of Palermo; 2 Department of Vascular and Endovascular Surgery, University Federico II of Naples (umbertomarcello.bracale@unina.it)

**Keywords:** coronary atherosclerosis, carotid artery disease, coronary computed tomography angiography, coronary angiography, duplex scan

## Abstract

The evaluation of coronary lesions in patients with asymptomatic carotid plaque represents a very promising line of research to assess cardiovascular risk and the possible implementation of a more aggressive prevention therapy.

**Methods:**

In this study we enrolled 102 patients with intermediate to high cardiovascular risk but no history of coronary artery disease. The first group, consisting of 51 patients, underwent a Coronary CT scan (CCT-group) as well as carotid ultrasonography. The second group, also consisting of 51 patients, underwent coronary angiography (CA) and carotid ultrasonography.

**Results:**

The absence of a statistically significant difference between the involvement of both coronary and carotid sites, assessed by CCT and CA, confirms the role of coronary CT as a useful method in the preclinical evaluation of cardiovascular risk. In the CCT group, the correlation between atherosclerosis of carotid artery and coronary disease, as well as between the mean carotid intimal medial thickness and the number of involved coronary vessels, and between the maximum values of carotid plaque and the presence of coronary artery stenosis > 50%, were statistically significant. The Agatson calcium score was also statistically associated with carotid plaque size.

**Conclusion:**

The imaging biomarkers have a key role in the evaluation of subclinical atherosclerotic disease. Moreover, carotid ultrasound examination and a CT-scan of coronary arteries, in a particular sub-group of patients with intermediate to high cardiovascular risk, can play a crucial role to assess the preventive therapeutic strategies.

## I. INTRODUCTION

Patients with increased cardiovascular risk benefit considerably from a stratification of this risk through the use of several markers (serum or imaging biomarkers) for subclinical atherosclerosis [1–3], despite the fact that the right therapeutic strategy is still a matter for discussion [4–8]. Several recent publications [9–11] indicate that this preclinical condition increases global cardiovascular risk. Therefore, the clinical application of the intimal-medial thickness (IMT) measurement and plaque identification can help to establish a pre-existent cardiovascular risk. The IMT was measured via a simple carotid duplex scan and is a useful and cheap tool to analyse the risk stratification in asymptomatic patients [12]. Moreover, patients with a family history of cardiovascular disease (men < 55 years old, women < 65 years old), patients under 60 years of age who demonstrate serious changes in a single risk factor, but who otherwise would not be candidates for pharmacological therapy, and finally women under 60 years of age with at least two risk factors, should also be subjected to IMT measurement and investigation for carotid plaques. Kablak-Ziembicka et al. [13] have reported their results on 588 subjects using ultrasonographic evaluation of the IMT of the carotid arteries. They correlated the IMT with the number of coronary diseased vessels (p=0.001) and the degree of stenosis, proposing this as a highly specific and sensitive indicator of multifocal atherosclerosis and advanced coronary involvement. In particular, the statistical analysis showed a high correlation between an increase in the IMT value at the level of the common carotid artery (CCA) and the presence of significant coronary stenosis (>50%). Over the last few years, the use of computed coronary tomography (CCT) has increased considerably, and has proved to be useful in an ever-widening category of patients [14–19].

This study attempts to assess the relationship between asymptomatic carotid atherosclerosis and coronary artery disease (CAD), evaluated respectively by use of duplex scan and CCT or coronary angiography (CA).

## II. METHODOLOGY

### Study population and method

We assessed patients admitted to our hospital over a two-year period (2011/2012) and recruited those who underwent a CT-scan of the coronary arteries, (chosen according to the current indications [21], that is after an inconclusive stress test result, or those who were unable to perform the stress test or were asymptomatic but with provocative test results indicative of ischemia). Each patient underwent analysis of the carotid profile through carotid ultrasonography according to the ASE protocol [22]. Due to technical problems, or a contraindication to the CT scan (atrial fibrillation, irregular heart rate or being > 65 years old although undergoing beta-blocker therapy) a second group of patients, with the same indications, underwent coronary angiography. The carotid profile was also evaluated in this group through carotid ultrasonography.

In this way, we formed two groups of patients, the CCT group, composed of 51 patients who underwent a carotid duplex scan and coro-CT scan, and the CA (coronary angiography) group, which was also composed of 51 patients, who were subjected to a carotid duplex scan and coronary angiography. Patients with eGFR< 30mL/min/1.73m2, or allergy to the contrast medium were excluded from the study.

Each patient was evaluated in terms of the main cardiovascular risk factors: cigarette smoking, arterial hypertension, diabetes mellitus, dyslipidaemia, obesity, and a family history of cardiovascular disease as well as in terms of current therapy. Each patient signed an informed consent form about the design and purpose of the study. The patients were subjected to an ultrasound duplex test scanning of the carotid common arteries along the longitudinal axis up to the bifurcation (Siemens Sequoia C512 with linear probe, sound of 7.5/10 MHz). IMT <0.9 (measured at the terminal part of the common carotid artery) was considered normal and atherosclerotic plaque was defined as a limited protrusion within the vascular lumen >1.5 mm.

For the execution of the coro-CT scan, a 128-slice Siemens SOMATOM Definition Edge was used. Before the examination, each patient underwent an electrocardiogram and heart rate check. In patients with a heart rate up to 65 bpm, propanolol (40 mg, 2 tablets/day) or metoprolol (100 mg, 1.5 tablets/day) was administered four days before the CT-scan. In some selected cases, we also administered 10–15 drops of diazepam.

The scan was performed with a retrospective ECG synchronized acquisition technique. The mean exposure dose during CCT examination was about 7.5 mSv. In each case the calcium score was obtained using the Agatson method. For the purposes of evaluation, a window between 60% and 70 % of the R-R interval was used. Some bi-dimensional reconstruction techniques with different kernel and FOV (MPR) and tri-dimensional (MIP, Min-IP, shaded surface display, or surface rendering, and Volume Rendering) were used.

### Statistical Analysis

Data are expressed as the average ± standard deviation (SD) for continuous variables and as absolute and relative frequencies for discrete variables.

The comparison of the continuous variables (i.e. calcium score, plaque size, IMT) between the two groups was performed using a two paired t-test and analysis of the variance. The dichotomous variables (i.e hypercholesterolemia, hypertension, diabetes, current smoking, obesity, familiarity) compared between groups were evaluated with the Mann-Whitney U-test. Data were correlated through uni- and multivariant analysis looking for statistically significant associations and through the constitution of ROC curves.

## III. RESULTS

**Figure 1:**
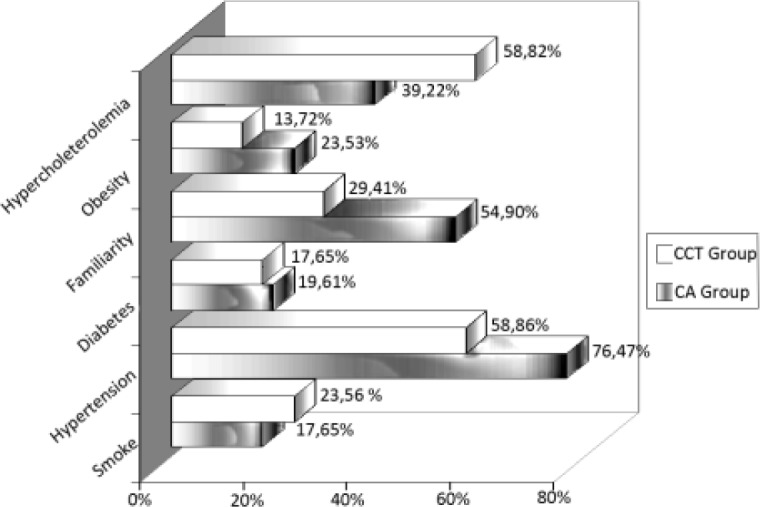
distribution of the cardiovascular risk factors

The CCT group was composed of 51 patients, 32 males and 19 females, with an average age of 57.3 years. The CA group was composed of 51 patients, 30 males and 21 females, with an average age of 58.2 years. The distribution of the cardiovascular risk factors is shown in Figure 1.

**Figure 2:**
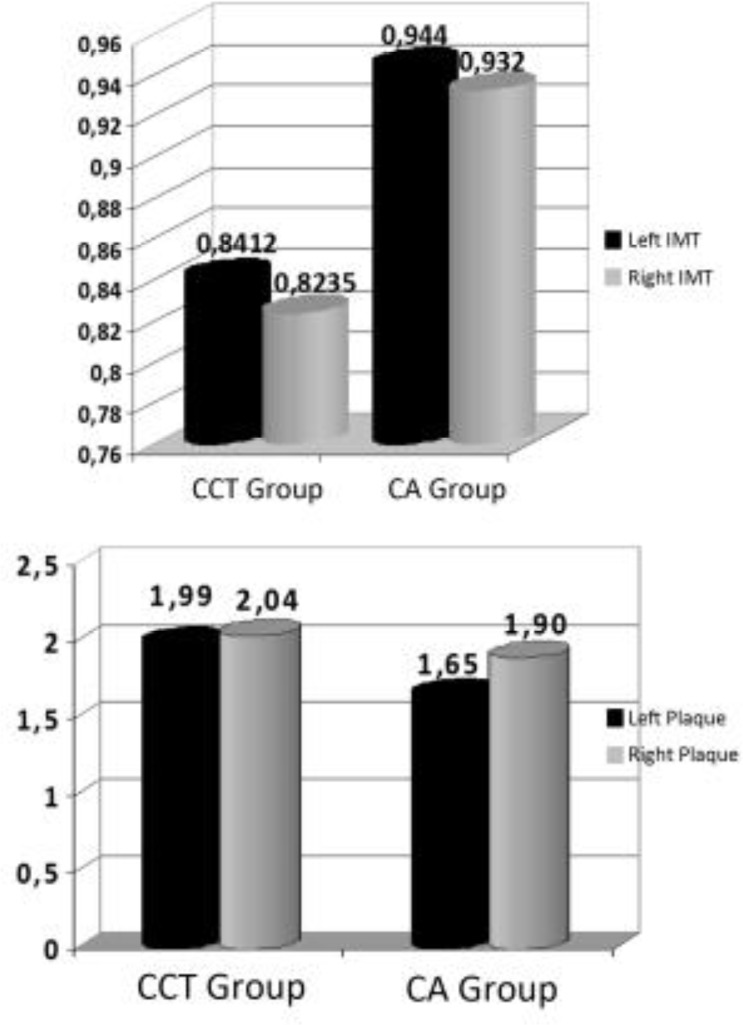
IMT and carotid plaques detected.

Patients were evaluated according to their current therapy, their cardiovascular risk and the possible presence of IMT and/or carotid plaques. Figure 2 shows data regarding the IMT values and/or carotid plaques.

Table 1 shows the coronary involvement, expressed for each segment, evaluated through coronary CT and divided in four classes: absence of stenosis, stenosis <50%, stenosis >50%, sub-occlusion/occlusion. Table 2 summarizes coronary involvement, for each segment, in the CA group. Comparing the two sites, the carotid and coronary ones, between the two groups, it can be seen that in 48% of CCT group cases (24 patients), there is a correspondence between the two locations in terms of the presence or absence of plaques, while in 52% of cases (26 patients) there is no correspondence. In the CA group, by contrast, the values are 44% (22 patients) and 56% (28 patients), respectively. The analysis of the comparison between the two sites is not statistically significant (p=0.964), thus indicating that both methods represent a good level of diagnostic capacity and of association between the two arterial regions.

Looking more closely at the two groups, we performed tests to evaluate possible statistical correlations among the main cardiovascular risk factors and preclinical atherosclerotic injuries in the carotid and coronary sites. This analysis revealed a statistically significant association between diabetes, hypertension and number of involved vessels and\or degree of stenosis >50%, as well as a correlation between both dyslipidaemia and smoking with average size carotid plaques (Table 3) We investigated the presence of a statistically significant correlation between the locations of atherosclerotic disease in carotid and coronary sites (table 4).

**Figure 3:**
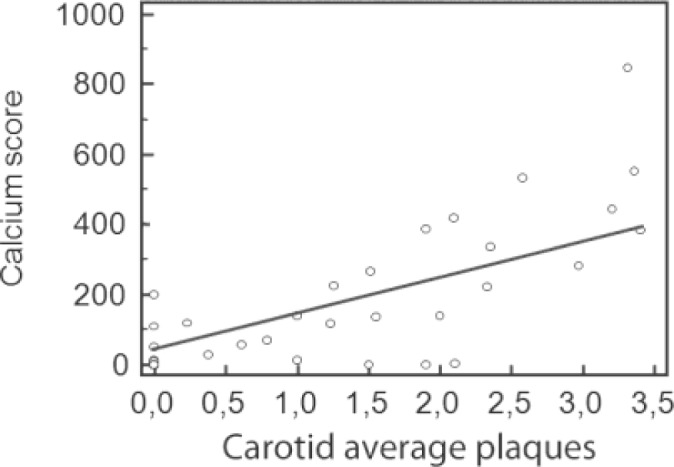
Correlation between Calcium Score and carotid average plaques

In particular, we noticed that in the CCT and CA groups, an increase in IMT and/or carotid plaque values is associated with an increase in the level of coronary plaque (p=0.023; OR=3.39). Both in CA and CCT groups there was a statistically significant association between the IMT average and the number of vessels with coronary plaques (p=0.04; OR=2.14) and also the average size of carotid plaques and the number of vessels with coronary plaques (p=0.004;OR=3.06). However, only in the CA and CCT groups is there a significant correlation between the greater values of carotid plaque and the presence of stenosis greater than 50% (p=0.0027; OR=0.8943) and between the greater values of carotid plaque and the presence of coronary plaques (p=0.006; OR=2.89). The correlation between Calcium Score and values of carotid plaques also appears statistically significant. (p=0.0048; OR=2.9958) (Figure 3). Using an ROC curve (table 5) it was revealed that for an IMT average of more than 0.9 mm it was possible to predict the presence of coronary stenosis evaluated through coronary CT.

## IV. DISCUSSION

Nowadays the methods for stratification of cardiovascular risk can also make use of the coronary CT technique. The non-invasive character of the method makes it of great interest to consider its use to define the quantity of coronary calcium, even if the role of the calcium ion remains unclear in the process of plaque fracture or as a recognized marker of asymptomatic atherosclerosis. The search for coronary fractures in patients with asymptomatic carotid plaque represents a potentially interesting possibility for the use of this method. It could be useful in order to carry out an evaluation of coronary history, of the carotid profile, and also to provide a multilevel atherosclerotic profile in order to assess an exhaustive determination of the global cardiovascular risk and to design a more aggressive prevention strategy [23]. The absence of a statistically significant difference (p=0.964) between the involvement of the two different sites, coronary and carotid, as evaluated through CCT and CA, confirms the role of coronary CT for use in the pre-clinical evaluation of cardiovascular risk. For the CCT group, the correlation between the presence and level of atherosclerotic disease in the coronary and carotid sites (p=0.01;OR=3.39) is statistically significant and supports the multi-level nature of the atherosclerotic process. The correlation of the IMT average or average carotid plaque size and the number of involved coronary vessels is also statistically significant (p=0.04; p=0.004), as is the correlation between high values of carotid plaque and the presence of coronary stenosis >50% (p=0.006). In our study, the calcium score according to Agatson is statistically associated with the dimensions of the carotid plaques (p=0.0048; OR=2.9958), but not with values of IMT (p=0.7440; OR=-0.3290), thus representing a more advanced atherosclerotic fracture (calcific plaques) indicator. Using CCT, the most sensitive (75%) and specific (64.29%) cut-off of IMT to predict the presence of coronary stenosis and degree was 0.9 mm which corresponds to the value established by the 2007 European Society of Hypertension- European Society of Cardiology (ESH-ESC) Guide-Lines regarding the management of patients with arterial hypertension (validated upon comparison with carotid Duplex scan and CA) [24].

Lim et al. [25] found that coronary atherosclerosis on coronary artery calcium (CAC) and CTA were similarly related to carotid disease. Carotid plaque, maximal IMT ≥1.5 mm and averaged mean IMT >0.75 mm predicted CAD independent of age and sex. Plaque had a closer relationship with CAD than maximal or average mean IMT, especially to CAC. CAD was present in most patients with carotid plaque or increased IMT (maximal IMT ≥1.5 mm or mean IMT >0.75 mm) and absent in most patients without carotid plaque or with lower IMT values. Moreover, Sillesen et al. [26], who examined subclinical atherosclerosis in an older population and found a stronger relationship between CAC and 3-dimensional carotid plaque burden than mean CCA IMT, found carotid plaque in more subjects (78% vs. our 71%) than a non-0 calcium score (68% vs. our 58%). Bauer et al. [27] found that carotid IMT was more closely related to diabetes, and CAC was more closely related to hypertension.

## V. CONCLUSION

In light of these data, it appears clear that CCT shows a very high sensitivity and specificity when compared with traditional CA for use in patients with intermediate to high risk or when there are uncertain results to the stress test or when it cannot be performed. The technical improvement to the CT scanners, that can significantly reduce the required radiation dose and increase temporal resolution, should allow them to emerge as an effective, non-invasive method in the approach patient care and in the stratification of the global cardiovascular risk.

## Figures and Tables

**Table I:** coronary involvement, expressed for each segment, evaluated through coronary CT, divided in the four classes: absence of stenosis, stenosis <50%, stenosis 50%, sub occlusion/occlusion.

**N°: 51**		**Absence of stenosis**	**Stenosis < 50%**	**Stenosis 50%**	**Stenosis >50%**	**Subocclusion/occlusion**
Right coronary arterty	Proximal	33(64.7%)	16(31.4%)	0(0%)	2(3.9%)	0(0%)
	Intermed.	36(70.6%)	10(19.6%)	2(3.9%)	3(5.9%)	0(0%)
	Distal	39(76.5%)	9(17.6%)	1(2%)	2(3.9%)	0(0%)
	PDA_2_	46(90.2%)	3(5.9%)	1(2%)	1(2%)	0(0%)
Left main coronary artery			9(17.6%)	0(0%)	0(0%)	0(0%)
LAD1	Proximal	24(47.1%)	18(35.3%)	6(11.8%)	2(3.9%)	1(2%)
	Intermed.	31(60.8%)	10(19.6%)	3(5.9%)	7(13.7)	0(0%)
	Distal	48(94.1%)	2(3.9%)	0(0%)	1(2%)	0(0%)
	I Diagonal	40(78.4%)	7(13.7%)	1(2.0%)	3(5.9%)	0(0%)
	II Diagonal	47(92.2%)	2(3.9%)	0(0%)	1(2.0%)	1(2,0%)
Circumflex	Proximal	38(78.5%)	7(13.7%)	0(0%)	5(9.8%)	1(2%)
	Distal	46(90.2%)	3(5.9%)	0(0%)	2(3.9%)	0(0%)
	I marginal	49(96.1%)	2(3.9%)	0(0%)	0(0%)	0(0%)
	II marginal	47(92.2%)	3 (5.9%)	0(0%)	1(2%)	0(0%)
	PDA	47(92.2%)	3(5.9%)	0(0%)	1(2%)	0(0%)
Intermediate Branch		50(98%)	0(0%)	1(2%)	0(0%)	0(0%)

1: Left anterior descending artery,

2: Posterior descending artery

**Table II:** coronary involvement, expressed for each segment, evaluated through coronary angiography, divided into the four classes: absence of stenosis, stenosis <50%, stenosis 50%, sub occlusion/occlusion.

**N°: 51**		**Absence of stenosis**	**Stenosis < 50%**	**Stenosis 50%**	**Stenosis >50%**	**Subocclusion/occlusion**
Right coronary arterty	Proximal	48(94%)	3(6%)	0(0%)	0(0%)	0(0%)
	Intermed.	45(88%)	6(12%)	0(0%)	0(0%)	0(0%)
	Distal	45(90%)	4(8%)	2(2%)	0(0%)	0(0%)
	PDA_2_	49(97%)	2(3%)	0(0%)	0(0%)	0(0%)
Left main coronary artery		0(0%)	4(6%)	0(0%)	0(0%)	0(0%)
LAD_1_	Proximal	42(84%)	5(10%)	4(6%)	0(0%)	0(0%)
	Intermed.	43(86%)	8(14%)	0(0%)	0(0%)	0(0%)
	Distal	48(94.1%)	3(5.9%)	0(0%)	0(0%)	0(0%)
	I Diagonal	48(96%)	1(2%)	0(0%)	0(0%)	1(2%)
	II Diagonal	45(90%)	2(4%)	1(2%)	0(0%)	1(2%)
Circumflex	Proximal	48(96%)	3(4%)	0(0%)	5(9.8)	1(2%)
	Distal	51(100%)	0(0%)	0(0%)	2(3.9)	0(0%)
	I marginal	47(94%%)	3(6%)	0(0%)	0(0%)	0(0%)
	II marginal	50(98%)	0(0%)	0(0%)	1(2%)	0(0%)
	PDA	50(98%)	0(0%)	0(0%)	1(2%)	0(0%)
Intermediate Branch		50(98%)	1(2%)	0(0%)	0(0%)	0(0%)

1: Left anterior descending artery,

2: Posterior descending artery

**Table III:** Correlation among the main cardiovascular risk factors and preclinical atherosclerotic injuries in carotid and coronary arteries

N° 102	Dibetes Mellitus		Hypertension		Hypertriglyceridemia		Hypercholesterolemia		Smoking Cigarettes	
	N° vessels CA/CT		N° vessels CA/CT		Stenosis >50% (CCT)		Carotid plaques		Carotid plaques	
Cases (CCT)	0.0285	2.26	0.03	2.16	0.0279	2.2667	0.2113	1.2666	0.5335	0.62
Controls (CA)	0.48	0.71	0.24	1.18	0.7221	0.3578	0.0188	2.4934	0.0050	3.06
	P value	Odds Ratio	P value	Odds Ratio	P value	Odds Ratio	P value	Odds Ratio	P value	Odds Ratio

**Table IV:** statistically significant correlation between location of the atherosclerotic disease in carotid and coronary arteries

N°: 102	IMT/ Carotid plaque		IMT		Carotid plaques		Carotid plaque		Carotid plaque	
	Level of coronary plaque		N° vessels with coronary plaques		N° vessels with coronary plaques		Stenosis >50%		Presence of coronary plaques	
Cases (CCT)	0.023	3.39	0.04	2.14	0.004	3.0578	0.0027	3.17	0.006	2.89
Controls (CA)	0.1842	1.35	0.001	2.68	0.02	2.5141	0.8943	0.13	0.69	0.39
	P value	Odds Ratio	P value	Odds Ratio	P value	Odds Ratio	P value	Odds Ratio	P value	Odds Ratio

**Table V:** Roc curve results that for average IMT more than 0.9 mm we have the great specificity and sensibility in predicting the presence of coronary stenosis evaluated through coronary CT.

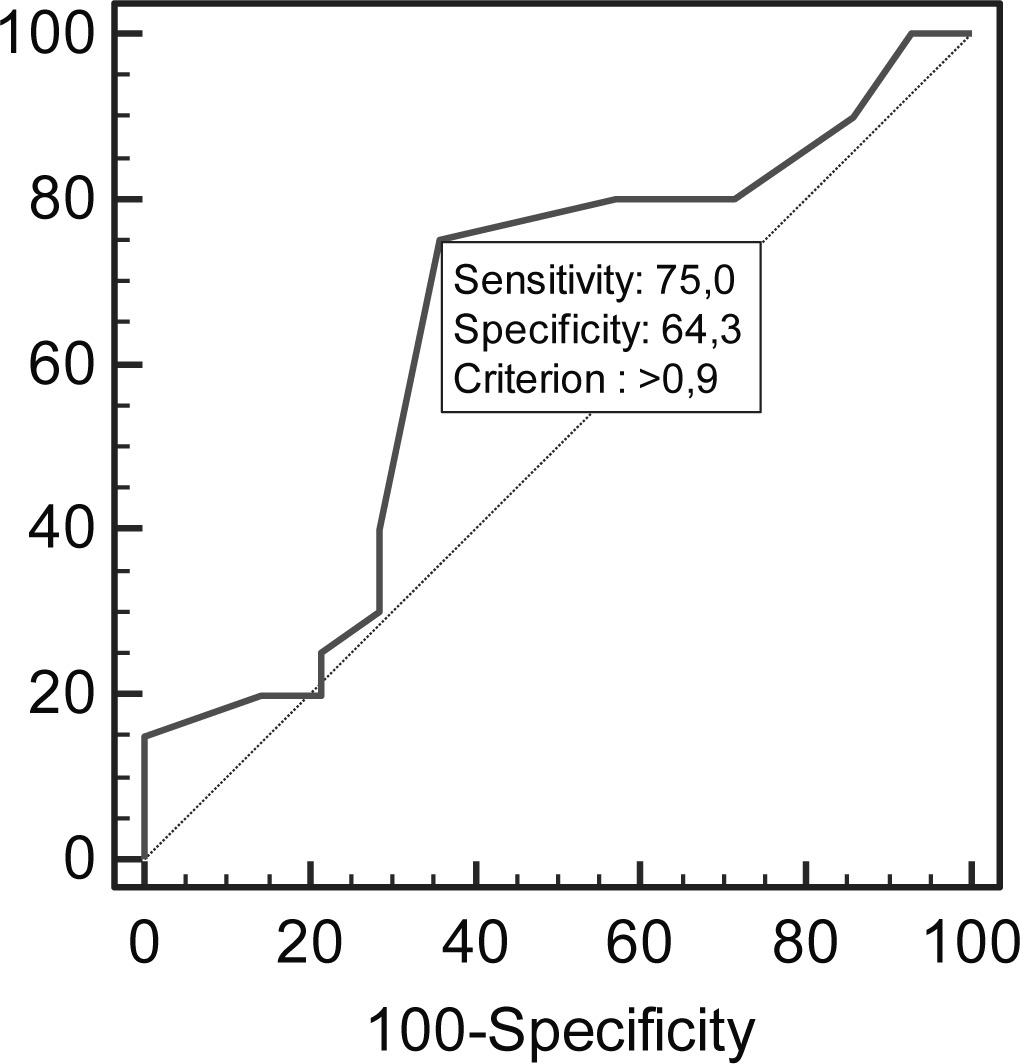				
**N°**	**Criterion**	**Sensitivity**	**Area under the curve**	**Specificity**
**51**	**> 0.9 mm**	**75%**	**0.641**	**64.29%**
